# Stigma resistance and quality of life in patients diagnosed with borderline personality disorder

**DOI:** 10.1192/j.eurpsy.2025.1480

**Published:** 2025-08-26

**Authors:** A. Gmeiner, A. Mochty, A. Wippel, M. Amering

**Affiliations:** 1 Social Psychiatry; 2 Comprehensive Center for Clinical Neurosciences and Mental Health, Medical University of Vienna, Vienna, Austria

## Abstract

**Introduction:**

Unlike data on public stigmatization with regard to the diagnosis of borderline personality disorder (BPD), literature concerning internalized stigma (IS) and stigma resistance (SR) is scarce.

**Objectives:**

The objective of the present study was collecting data about SR in patients diagnosed with BPD. Furthermore, quality of life and depressive symptoms were assessed and checked for possible associations with SR.

**Methods:**

Patients meeting the inclusion criteria with 18-65 years of age and diagnosed with BPD were included into the study after giving written informed consent. The stigma resistance scale (SRS) had been translated to German. The SRS comprises 5 subscales and measures SR on a 5-point-Likert scale (1-5). Higher values indicate higher levels of SR. Furthermore, the German versions of the depression scale CES-D (ADS) and the quality of life – BREF (QOL-BREF) were used in this study. ADS measures depressive symptoms on a scale ranging from 0-60, with higher scores indicating more severe symptoms. QOL-BREF measures values between 0-100 on 4 domains, with 100 indicating a maximum of health in each domain. The recruitment took place in specialized inpatient and outpatient units between 2019 and 2022, with some interruptions caused by COVID protective measures.

**Results:**

101 participants (f = 90.1%) with a mean age of 29.2 years (18-59) were included into the study. The overall mean score of the SRS was 3.6. The mean scores of the subscales showed values between 2.8 (personal cognition) and 3.9 (public SR). The 4 domains of the QOL-BREF showed the following mean values: Physical health 47.4, psychological health 30.7, social relationships 51.2, environment 62.1. The study sample had a mean ADS score of 34.8. Interestingly stigma resistance, quality of life and depressive symptoms showed no correlation. Compared to the mixed sample in the original study, the sample of the current study showed comparable values in stigma resistance.

**Conclusions:**

The use of the German SRS is feasible in a sample of patients with a diagnosis of BPD and yielded comparable results with the original mixed diagnoses study sample. As the measure exists already in several languages, comparisons between cultural, diagnostic and otherwise defined subgroups will be possible in the future. Further conceptual as well as qualitative and quantitative research work will be informed by these findings.

**Disclosure of Interest:**

None Declared

